# The History of Bovine Genital Campylobacteriosis in the Face of Political Turmoil and Structural Change in Cattle Farming in Germany

**DOI:** 10.3390/vetsci10120665

**Published:** 2023-11-23

**Authors:** Hosny El-Adawy, Helmut Hotzel, Herbert Tomaso, Heinrich Neubauer

**Affiliations:** 1 National Reference Laboratory for Bovine Genital Campylobacteriosis, Institute of Bacterial Infections and Zoonoses, Friedrich-Loeffler-Institut, 07743 Jena, Germany; 2 Institute of Bacterial Infections and Zoonoses, Friedrich-Loeffler-Institut, Federal Research Institute for Animal Health, 07743 Jena, Germany; h-hotzel@t-online.de (H.H.); herbert.tomaso@fli.de (H.T.); 3 Faculty Medicine of Veterinary, Kafrelsheikh University, Kafr El-Sheikh 35516, Egypt

**Keywords:** *Campylobacter fetus* subsp. *venerealis*, bovine genital campylobacteriosis, cattle breeding, Germany, Animal Health Law

## Abstract

**Simple Summary:**

*Campylobacter fetus* subsp. *venerealis* (*Cfv*) is the causative agent of bovine genital campylobacteriosis (BGC), also known as bovine venereal campylobacteriosis, which is a disease relevant to international trade listed by the World Organization for Animal Health (WOAH). In Germany, BCG was already found to be endemic in the 1920s, shortly after the agent and the disease had been described for the first time. It can be assumed that BCG had already circulated uncontrolled for a long time in the predecessor states of Germany, influenced only by the political situation and trading networks of the time. A single genetic *Cfv* lineage was identified which probably emerged in the 19th century and diversified over time. Interestingly, no recurrent cross-border introduction became evident. Recent ambitious efforts of German agricultural policies made for animal welfare, conservation of environment and genetic resources and necessary structural adjustment must go hand in hand with control measures of veterinary public health. This review gives insight into the history of bovine genital campylobacteriosis considering the structural change in cattle farming in Germany and reflecting on the political background of the time.

**Abstract:**

Contagious bovine genital campylobacteriosis (BGC), also known as bovine venereal campylobacteriosis, is a disease relevant to international trade listed by the World Organization for Animal Health (WOAH). It is caused by *Campylobacter fetus* subsp. *venerealis* (*Cfv*), one of three subspecies of *Campylobacter fetus*. Bulls are the reservoir but BGC may also be spread by artificial insemination (AI). BGC is characterized by severe reproductive losses such as infertility, early embryonic death and abortion with considerable economic losses. This significant economic impact has prompted several countries to adopt stringent eradication and surveillance measures to contain the disease. While there are commercial and autologous vaccines available, scientific evidence for the effectiveness of vaccination is still lacking. In Germany, BCG was already found to be endemic in the 1920s, shortly after the agent and the disease had been described for the first time. It can be assumed that BCG had already circulated uncontrolled for a long time in the predecessor states of Germany, influenced only by the political situation and trading networks of the time. After WW II, BCG was eradicated in the German Democratic Republic due to industrialized cattle production based on AI but it was still endemic at low levels in the Federal Republic of Germany with its diverse cattle production. There has been a steady decline in BGC incidence in re-unified Germany over the past 28 years. A single genetic *Cfv* lineage was identified which probably emerged in the 19th century and diversified over time. Interestingly, no recurrent cross-border introduction became evident. This review gives insight into the history of bovine genital campylobacteriosis considering the structural change in cattle farming in Germany and reflecting on the political background of the time.

## 1. Introduction

*Campylobacter fetus* subsp. *venerealis* (*Cfv*) is the causative agent of bovine genital campylobacteriosis (BGC). Severe reproductive losses resulting from infertility, early embryonic death and abortion were leading to strict national control programs and monitoring of international trade of cattle, sperm, oocytes and embryos [1,2,3]. The incidence of BGC is highest in low- and middle-income countries (LMIC), where natural breeding of cattle is widely practiced, when compared to high-income countries, where cattle breeding benefits from strict control of bulls and sperm used for artificial insemination (AI) [4]. The often clinically inapparent bulls are a reservoir for spreading the infection. Cows become infected during natural service or after AI with contaminated semen. Bulls can become infected by serving an infected cow and transmission may occur between bulls during mounting. Vertical transmission has never been reported. BGC infections in cows are usually self-limiting and most cows usually regain fertility within 5 months following elimination of the infection from the uterus. Bulls can be lifelong carriers of the pathogen [3]. BGC is diagnosed by diagnostic tools prescribed by the World Organisation of Animal Health (WOAH) [1,2]. Isolation of *Cfv* can be challenging, since it is slow-growing and requires specific microaerobic conditions. Identification of *C. fetus* can be done with biochemical tests or molecular tests. BGC is controlled by vaccination, antimicrobials, artificial insemination and quarantine measurements, although the effectiveness of vaccination is questioned [1,3].

The *Cfv* population was shown to be genetically conserved but has evolved in geographic clusters [5]. *Cfv* strains from Germany collected between 1985 and 2015 were recently assigned to a single cluster that may have emerged around 1840 [6]. This unexpected timing of the emergence of the German *Cfv* cluster at a time of profound changes in European history prompted us to investigate the fate of BCG in Germany reflecting the interactions of the rapid change in political systems, the development of modern infectiology, the structural change in agriculture and cattle breeding and customer demands and recent attitudes towards animal welfare.

## 2. A Short Excursion into Recent ‘German’ History

The emergence of the ‘German’ cluster of *Campylobacter fetus* subsp. *venerealis* coincides with the ‘Deutscher Bund’ (1815–1866). This confederation was a loose association of sovereign state entities with large kingdoms like Prussia or Bavaria, parts of the Austrian empire, Danish Holstein and Dutch Luxemborg but also free cities like Hamburg or Bremen. The area stretched from the North and Baltic Seas to the Mediterranean Sea and from the borders of the kingdoms of France, the Netherlands and Belgium and the Swiss Confederation to those of the kingdoms of Russia, Poland and Hungary (https://de.wikipedia.org/wiki/Deutscher_Bund, accessed on 2 March 2023). This area was also, later on, often referred to as ‘Germany’ in the literature used for this review, although this simplification was never reflecting the true political situation or actual trade networks. It has to be stressed that ‘Germany’ changed its appearance fairly often due to the numerous wars in Europe in the following centuries, and our statements relate to the respective borders valid at the time concerned (German Empire 1871–1918, Weimarer Republic 1918–1933, German Reich 1933–1945, Allied-occupied Germany 1945–1949, German Democratic Republic (GDR, 1949–1990) and Federal German Republic (FRG, 1949–….)).

## 3. *Campylobacter fetus* Subspecies *venerealis*, a Quiet but Steady Companion of (German) Cattle Breeding

Before 1913, infectious abortions in cattle were generally attributed to brucellosis i.e., infection with *Brucella abortus*. Then, Gram-negative (microaerophilic), comma-shaped bacteria were discovered to be causing abortion in sheep and cattle in the UK [7] and US [8] and were called *Vibrio fetus*. These bacteria were subsequently also isolated in 1920 in Denmark [9] and in 1922 in the German states of Wuerttemberg [10] and Saxony [11]. At that time, it can be assumed that ‘*Vibrio fetus*’ was already distributed over all European countries and their global trading partners, considering the restocking campaigns after the disastrous Rinderpest outbreaks of the 19th century and the steadily continuing transboundary trade and movement of meat, breeding and draft cattle of previous centuries or even millennia [12]. In 1959, Florent proposed to collect all *V. fetus* strains restricted to the uro-genital tract of bovines causing enzootic abortion in the subspecies *V. f. venerealis* (no growth on agars containing 1% glycine or H_2_S cysteine) and gut-associated strains causing sporadic abortion in the subspecies *V. f. fetus* [13]. In early German literature, the name *Vibrio fetus* type 1 was often applied to *Cfv* strains [14]. Later on, these *Vibrio* species were reclassified as *Campylobacter fetus* [15] and the disease caused by *Cfv* became known as bovine genital campylobacteriosis (BGC) [2]. BGC is transmitted only during mating or via contaminated sperm and equipment used for artificial insemination, e.g., artificial vagina, pipettes, gloves, etc. Bulls may be infected when mounting each other or via contact to surfaces contaminated with genital secretions. *Cfv* colonizes the mucous membrane of the male genital tract without causing lesions or disease but causes lifelong infection [16]. In cows, infections provoke infertility, early embryonic death and abortion but predominantly extended calving intervals are responsible for the economic losses [17]. The cows become clean again and may give birth to healthy calves again but may be re-infected when immunity decreases. In a herd with natural mating a balance may develop, and only the first breeding and newly introduced cows may abort. New bulls get infected by older cows instead [16]. Herds can become chronically infected and eradication of BCG can last several years if no harsh countermeasures are applied. The disease can be easily spread [18]. Data from Argentina and Australia suggest that the weaning rate may decrease by 10% and the gross profit margins may be reduced by 66% and 36% in the first and the following years, respectively [3].

The gold standard in diagnosis is still the cultivation of the agent from preputial samples of bulls (scraping, suction, or washings), cervico-vaginal mucous samples from cows and heifers, or samples from aborted fetuses (abomasal content, lungs, liver and placenta) and subsequent phenotyping or genotyping. Especially in chronically infected bulls, cultivation is troublesome but always needs specialized laboratories and experienced personnel.

Serological assays are not specific and are not advised to be used for definitive diagnosis. Several commercial vaccines are available for BGC consisting of inactivated *C. fetus* cells. Both male and female cattle can be vaccinated against *C. fetus*. It is recommended to vaccinate against BGC by annual revaccination with a single dose between 30 days and 7 months before breeding. Vaccination of bulls might help to control the spread of infection but the effect is limited [3].

Treatment of bulls e.g., with streptomycin or oxytetracyclines locally can be successful if the bulls are less than 3 years old, while antibiotic treatment of older bulls is often not sufficient to clear the infection, and the older bulls remain lifelong carriers [19]. The effectiveness of antibiotic treatment in cows and heifers is unknown, since female cattle are not usually treated because treatment results are poor and most females develop protective immunity, enabling them to resist re-infection [20,21].

Proper husbandry practices reduce the risk of introducing *C. fetus* into a herd. The major biosecurity measure against BGC is the use of BGC-free bulls, semen, or embryos. This will ensure that BGC is not introduced into a herd. In the EU, Council Directive 88/407/EEC, which was replaced by EU Animal Health Law EU2016/429 later on, did set the measures for the animal health conditions of intra-community trade and third-country imports of frozen bovine semen. Testing for *C. fetus* infection should be carried out (either by IFAT or by culture) in animals of approved semen collection centers [3].

Culling of bulls and cows is controversial and needs careful evaluation. With regard to the monitoring of free-ranging herds, the precise examination of material from all aborted fetuses and the development of meat juice ELISAs will therefore become increasingly important in the future. In the FRG and GDR, bovine venereal diseases became notifiable in 1975 and BCG is controlled by continuously testing mating bulls at artificial insemination centers. As BGC has significant economic implications, stringent regulations for the trade of cattle, sperm, embryos and oocytes are in force. Thus, a low incidence of BGC outbreaks (*n* = 68 since 1994) in nine of sixteen federal states has been guaranteed. Outbreaks are caused by an endemic, genetically conserved German *Cfv* lineage with two major lineages (German Lineage 1 [northern states] and German Lineage 2 [southern states]) [6]. This distribution is in line with the distribution of German cattle breeds, i.e., specialized dairy cattle (German Black Pied cattle, lowland cattle) in northern Germany and mixed-purpose cattle (Höhenvieh, highland cattle) in southern Germany [22]. It has to be noted that *Cfv* strains originating from the former GDR and FRG both belong to the German lineage, pointing to a common ancestor before 1945, and before the ‘iron curtain’ (the physical barrier between both German states made with minefields, fences, guards and concrete) was built in 1961 and trade therefore stopped [6]. It is noteworthy that no introduction of *Cfv* strains from endemic countries has been documented by genetic investigation until now, which shows the effectiveness of the regulations in force.

German strains are susceptible to gentamicin but reduced susceptibility was noted, mostly for lincomycin and spectinomycin, in 14% of strains [23]. In contrast, *Cfv* strains may be resistant to nalidixic acid, doxycycline, tetracycline and even ciprofloxacin and enrofloxacin. As a consequence, today any German *Cfv* isolate has to be tested before treatment of infected bovines is started.

## 4. The Wings of Change in Agriculture: Industrialization of Cattle Breeding

Before 1850, cattle breeding was subject to local taste and local needs, and a plethora of different races did exist in Germany which were fairly often crossed with breeds from other countries, e.g., the UK, Austria, or Switzerland to improve profit [24]. In the northern German and Dutch (Frisian) lowlands with abundant grassland, red and colored (pied) cattle were bred but black/white and red/white colored cattle (schwarzbunte (sb) and rotbunte (rb) Niederungsrinder) were becoming predominant due to excellent milk and meat production. These breeds have the taurine haplotype Y1 and may still represent the Danubian immigration of western Asian people with their cattle to northern Europe and the dairy breeding preferences of Germanic tribes in the past [22]. In northern Germany, breeder associations were not founded before 1868, and at that time the German sb with superior milk production was spreading all over northern, middle and eastern Germany [24,25]. After the second world war (WW II), the very productive sb breeding areas of eastern Prussia were no longer available [24]. As early as 1621, 1795, 1810, 1825 and from 1861 onwards, Dutch sb cattle were regularly imported to the USA and Canada and independent breeds were formed, i.e., Holstein and pure-bred Dutch Frisian cattle, which finally became the Holstein-Frisian (HF) breed in 1885 [25]. In the low mountain range of middle Germany, light, red/yellow colored breeds (Rotvieh/Gelbvieh) of different pedigrees were widespread and in the alpine south of Germany originally Swiss breeds, e.g., Simmentaler Fleckvieh (fv) or Braunvieh (bv), were widespread [24]. The yellow Franks (Gelbe Franken) were cattle bred for the forest pastures of Thuringia [24]. These undemanding and durable breeds best met the requirements for draft power and milk and meat production as the low mountain and alpine regions lack productive pastures and soils and have drier climates. Hence, lower yields in milk quantity, milk fat and protein contents had to be accepted [24]. These breeds have the taurine haplotype Y2 and may still reflect the Mediterranean immigration of Asian settlers but also the Romans’ preferences for draft and beef animals [22]. The intensive breeding of cattle was always connected with intensive cattle trade. For 1902, 238,335 cattle (breeding and slaughter) were imported and 8647 listed for the German customs area [26]. In the southern states of Bavaria and Baden Wuerttemberg and the southern part of middle Germany (Thuringia, south Saxony, south Saxony-Anhalt), the German fv became predominant before the 1930s [27]. It has to be noted here that a notable variety of breeds still remained with the smallholder-dominated cattle production of Germany. Hansen, in 1921, described 12 lowland and 24 indigenous cattle breeds of the mountainous regions of Germany [28]. Natural mating was the main practice at that time and outbreaks of venereal diseases were frequent in the so-called ‘Deckringe’ or ‘Deckbezirke’, i.e., cooperative organized by cattle farmers using common mating bulls [18,29]. Starting from a chronically infected Deckbezirk, BCG was easily spread to another by trade of infected bulls and cows, by ‘testing’ of bulls before purchase on the farm of the potential buyer and giving shelter to not in-house animals [18]. Therefore, regulations against venereal diseases, especially against the widely distributed trichomoniasis, had to be set in force in the late 1930s [30]. These regulations were later the basis for also combating BGC with amended regulations in both post WW II German states, the FRG and GDR. Hence, it has to be stressed that in both of the German states, formal regulations on *Cfv* were not set into force before 1975. With the advent of artificial insemination (AI) in the 1940s [24], additional facts had to be taken into account, i.e., many insemination bulls at the AI stations were infected at that time and could easily spread venereal infections with semen to various herds [31]. Wohanka, in 1957, described this new situation in depth and listed the statuary demands for the industrialized cattle production of the GDR. He was also the first to advocate for global hygiene standards [31]. For the FRG, Mitscherlich and coworkers summarized again the countermeasures to be taken if natural mating was applied. He also stressed the need for the use of AI and the use of insemination bulls that have tested negative for BCG to combat bovine venereal diseases effectively [18,32]. At that time, it also became obvious that *Campylobacter* can survive deep freezing and even the use of antibiotics in semen portions is not 100% effective [32,33]. Thus, regulations had to be amended accordingly over time for local and global trade [1,2].

Indeed, artificial insemination and N_2_ conservation of sperm has revolutionized cattle breeding and production in the 1960s and strict controlling of the bulls used for AI, e.g., quarantine, testing before and repeated testing during use were introduced to guarantee freedom of BCG. These countermeasures are still the strongholds of current European Animal Health Law and the statutory importance of BCG control has recently been stressed again (https://ec.europa.eu/food/animals/animal-health/animal-health-law_de (accessed on 8 November 2023)) [34].

It is worthwhile to analyze the cattle breeding systems in both of the post WW II German states in more detail for (current) risk analysis. From the 1950s onwards, the cattle production in the western FRG and eastern GDR followed different strategies. Hence, in both of the German states machine power replaced draft animals and physical power was no longer pursued as a breeding goal after 1960. Efficiency in increased milk and beef production was the new focus [24,35]. In the Soviet occupation zone, landowners with more than 100 hectares were expropriated in 1945 and small farms with 5 to 10 hectares were formed. This attempt at a smallholder system failed and from 1952 onwards agriculture of the socialistic GDR was restructured quickly. Industrialized, big agricultural cooperatives of farmers (Landwirtschaftliche Produktionsgenossenschaft, LPG) and state-owned enterprises (Volkseigene Betriebe, VEB) were formed for standardized production environments.

In 1950, 3.6 Mio cattle including 1.6 Mio cows and, in 1986, 5.8 Mio cattle (54 heads/km^2^) with 2.0 Mio cows were kept, respectively [27]. In 1961, a physical barrier, the iron curtain, was built by the GDR, preventing free movement of men, animals and goods for the next 29 years and effectively cementing the divergence of both of the German states. As a consequence of the mass production environment for industrialized cattle, only four cattle breeds were left in 1968 (sb, 87.4%; fv, 10.6%; yellow and red cattle, 1.8%; and 0.2% mixed cattle). Dutch sb bulls were introduced for breeding and sperm was imported from Canada and the USA [36]. In a next step, sb was re-bred using Danish Jersey, British Frisian and Holstein-Frisian (HF) cattle but also sb cattle from West German Ostfriesland [25,27]. Sperm of HF was imported from different states of the Council for Mutual Economic Assistance (Comecon, an organization of eastern European socialistic states that was operating globally from 1949–1991) to improve breeding results. Finally, the ‘schwarzbunte Milchrind’ (sbmr) delivered 3973 kg of milk per year with 4.02% fat and was the main cattle breed of the GDR in 1986. The lack of feed availability limited further breeding efforts. Fleckvieh (fv) breeding aimed at increasing milk production, and again Jersey bulls were used from 1961 onwards to improve yield but later on the HF was introduced. Also, a fv beef pedigree (fvb) was developed starting in 1971. In a farm housing 33,000 beef cattle, breeding experiments for 4 years with Chianina, Charolais, Marchigiana, Romangnola, Piemontese, Blue Belgian, Limousin, Herford, fv, South Devon, Sussex, Lincoln Red, HF, Jersey and sb bulls were done to optimize breeds for the needs of the GDR [37]. Sb, fv, Charolais (import of cows), Chianina (import of cows and bulls in 1975 from Italy) and Piemontese cattle (import of sperm and bulls) were kept in small numbers. Limousin cattle were imported from Hungary (bulls) and cows from France in 1986 [27]. The same author stated that the GDR had the opportunity to use the resources from the SSR (Socialistic Sowijet Republics) and Hungary earlier [36]. In 1986, 26.3% of the cattle kept were beef cattle [27]. In 1954, most of middle German (today the area of federal Saxony) ‘Deckringe’ was proven to be endemic for bovine vibriosis [38,39]. AI as a prerequisite for the industrialization of cattle production—production farms often had to house several thousands of heads—was indeed connected with a sharp decline in abortions. AI was already the usual practice in the 1950s, by 1960 it was practiced on up to 80% of the cows [39]. Finally, nearly 90% of cows were artificially inseminated in 1966 [40]. Trichomoniasis and BCG were considered to be more or less eradicated a decade later [39,41]. From 1965 to 1989, no case of *Cfv* infection was officially noted for the districts of the GDR (Tierseuchenberichte des Ministerialrates der Deutschen Demokratischen Republik, Landwirtschaftsrat, Abteilung Veterinärwesen, Berlin-Karlshorst 1965–1998). However, sporadic cases of *Cff* infections in bulls were still recorded [42]. Official trading partners of the GDR were considered to have high herd prevalences of *vibriosis genitalis* in 1954 and reported outbreaks later on, e.g., in the UK BCG was considered an infrequent but persistent problem for cattle breeders in 2000 [17,43]. Besides the few occasions connected to an official import that may had led to the introduction of *Cfv*, the smuggling of sperm from the FRG into the GDR was documented as well, in order to save the Vogtländische Rotvieh from extinction. Obviously, few private-run enterprises survived 40 years of agricultural industrialization [44]. The question of interest for risk analysis is whether *Cfv* could have also survived in such a controlled and nearly closed system of trade and production. Indeed, some strains of *Cfv* isolated from 1985 to 1988 in the GDR were sent to the new German reference laboratory after re-unification from Thuringian laboratories and showed a unique genetic pattern which was not found in the re-unified FRG again [6,45]. This GDR specific pattern may have been eradicated with the very fast changeover from the sbmr to imported HF. From 1993 onwards, the western FRG *Cfv* patterns were also found in the eastern FRG [6]. As the GDR strains were of German lineage, an introduction from import countries seems unlikely and BCG had obviously survived in the GDR.

In contrast to eastern Germany, a free-market economy developed in western occupation zones step by step. In 1950 11.15 Mio cattle (including 5.73 Mio milk cows), in 1960 12.9 Mio cattle (5.8 Mio) and in 1989 14.9 Mio cattle (60 heads/km^2^) (4.9 Mio) were kept in the FRG, respectively. A variety of cattle breeds did still exist in the early FRG [24,35,46]. Among other breeds, German shorthorn, German fv, German Braunvieh (in Baden-Württemberg and Bavaria, Allgäu), Grauvieh, Rotes Höhenvieh (pedigrees of the Southern red cattle) and Angler (red cattle of the peninsula Angeln, Schleswig-Holstein), gelbes Höhenvieh, German Angus (cross-breed of Aberdeen Angus with German breeds), Ansbach-Triesdorfer (in Ansbach, Bavaria, i.e., a multi-bred race from the beginning of the 18th century), Hinterwälder and Vorderwälder (in the black forest, Baden-Württemberg, land races), Limpurger and Glan cattle (on Limpurg Hills, Baden-Württemberg and in the Eifel/Hunsrück, Rheinland-Pfalz/Nordrhein-Westfallen, pedigrees of Gelbvieh), Murnau-Werdenfelser (in Murnau, Bavaria), Pinzgauer (in Southern Bavaria), were (and are still) kept [24,35,46,47,48].

In 1954, 52,700 mating bulls and 5,777,000 dairy cows were registered in the FRG, of which 1,564,000 were still used for draft [49]. Milk production was especially favored and the sb should therefore produce 5000 kg of milk per year with 4% fat [25]. As a consequence, the Holstein-Frisian breeding stock, semen and embryos were imported from North America starting in 1965 in order to increase the amount of milk. Finally, the ‘German Holstein’ (DH), with few genetic differences from the American Frisian-Holstein, was predominant in the FRG milk production industry and eventually replaced the sb cattle. The ongoing trend from smaller holdings to farms with more heads became a must and reflected the demographic change and the need to stay competitive in the local and global markets. Later on, sideline farming, hobby cattle husbandry and the economical need for diversification of the product panel favored keeping breeds like Charolais, Limousin, Jersey, Aberdeen Angus, Scottish Highland, Galloway, Salers, Aubrac, buffaloes/bisons, etc. [50]. These breeds were, and are, often kept in smaller herds with mating bulls. It can be assumed that in 1950 BCG was endemic in most, if not all, western German states, although only a few data are available for Lower Saxony (50% of herds tested), Bavaria, Schleswig-Holstein, Hessen and Nordrhein-Westfalen in 1958 [17]. In contrast to the state-dominated agriculture of the GDR and as a consequence thereof, the number of voluntary AIs was not higher than 30% in 1959 in the FRG, with noticeable local differences, e.g., 8.6% in Ostfriesland but 89% in Lübeck [24]. Hence, the number of AIs was slowly but steadily increasing and only sporadic notifications of ‘Deckseuchen des Rindes’ (venereal diseases of cattle), i.e., trichomoniasis, bovine vibriosis and infectious pustular vulvovaginitis, were registered after 1975.

It has to be noted that the consequent application of the Rinder-Deckseuchen-Verordnung of 1975 was an efficient tool to control widespread BCG. Finally, by 1981 BCG was still an endemic but sporadic disease in Baden-Wuertemberg [51,52]. As a consequence of this ordinance, the number of mating bulls was significantly reduced by the farmers as the benefits of AI, i.e., biosafety, labor saving and avoiding of dangerous bulls on the farm, were recognized and adopted quickly. It can be assumed that the situation described by Dedié and coworkers was also true for the other federal states. These authors also speculated that the restocking and intensified trade following the eradication of bovine tuberculosis and brucellosis in the 1950s and 1960s, and the strict testing and culling policy for trichomoniasis later on, had previously resulted in a fast and erratic spread of BCG in Germany.

The annual ‘Tierseuchenberichte der Bundesrepublik Deutschland’ reveals that only a few statements on causative agents were made and no metadata are available today. No *Cfv* strains are available for this time period from the FRG states for genomic analysis. Thus, retrospective epidemiological assessment of BCG is difficult but, as in the UK and other western European countries, BCG remained an infrequent but persistent problem for cattle breeders.

In 1990, the GDR and FRG became re-unified and again a deep change in agriculture was needed. The former GDR system of state-controlled production was abolished. East German farmers had the freedom and the economical need to react to the new markets, thus the sbmr of the GDR was replaced by the HF breed very fast in the new federal states that were formerly the GDR [53]. The sbmr and bfv breeds of the GDR (cross-breed fv with Charlois to get Uckermärker, race since 1992) breeds survived the structural upheavals and are still mainly kept in the eastern federal states [47]. These recent decades also saw a steadily increasing number of demographic, ethical and socioeconomic needs that forced and will force politicians and farmers to adopt substantial new strategies in animal production. Milk and beef production were, and still are, the main focus of cattle farming in Germany. The red/white and black/white colored breeds (red (r) and black (b) Holsteins) finally formed the Deutsche Holstein (DH) breed in 1995, and were intended to produce 10,000 kg of milk per year with 4.0% fat and 3.4% protein [25]. German fv (8200 kg milk, 4.2% fat, 3.5% protein) and Braunvieh (7800 kg milk, 4.29% fat, 3.64% protein) became the main dual-purpose cattle (https://www.rind-schwein.de/brs-de/statistik-zahlen.html, assessed 27 January 2023). In 2013, Brade and Brade listed 59% bDH, 8% rDH, 6% Braunvieh, 25% fv, 2% beef and 2% other races of herdbook animals (Table 1) [25]. The distribution of cattle in the 16 federal states of Germany is shown in Table 2.

**Figure 1:**
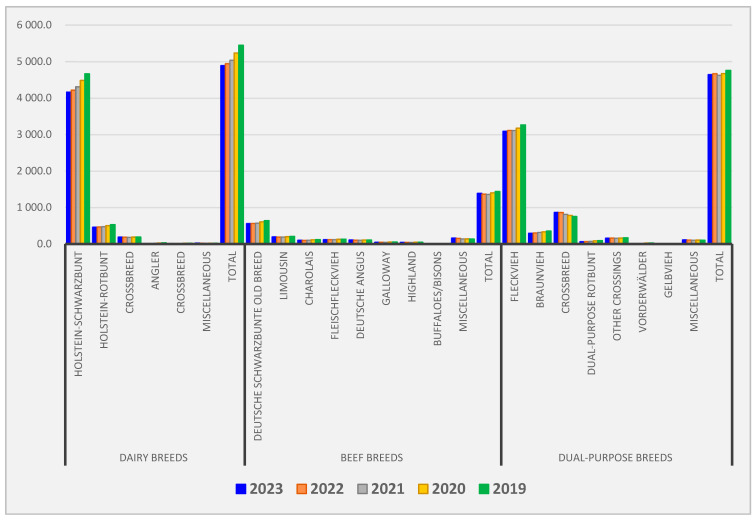
Distribution of cattle breeds by type of production and cattle breeds during the period 2019–2023 kept in Germany according to the Federal Agency for Agriculture and Food, The Federal Ministry of Food and Agriculture [48].

Around 11.3 million cattle were kept in 2022, including 3.9 million milking cows. In 2021, 4.8 million HF cattle and 3.2 million fv were kept but Angler, dsb, Limousin, Charolais, beef fv, German Angus, Galloway, Highlands, Braunvieh, rb, Vorderwälder and Gelbvieh were still kept in statistically relevant numbers. It has to be noted that 11,200 buffaloes and bisons were also registered, and approx. 165,000 thousand cattle fall under the category ‘others’, i.e., other breeds with insignificant numbers kept (Figure 1) [48].

Currently, 28 AI stations serve the needs of cattle production. Cattle and their products are and will be one mainstay of agriculture in the FRG. In 2021, approximately 25% of agricultural production value was rendered by cattle production, i.e., 14.3 billion euros. Germany was the biggest milk producer of the EU with 33,165 thousand tons of milk in 2020 (8457 kg per cow) and ranked second in beef production after France (1.090 thousand tons slaughter weight). The mean beef consumption per inhabitant is 9.81 kg. Hence, the production of drinking milk is declining (2021: 7.6 billion vs 2003: 8.9 billion liter) and the per head consumption was 47.8 kg in 2021, which is the lowest value since 1991 [54]. The consumer expectation of animal-friendly production close to nature led to very pleasing developments. In 2020, organic cattle farming was practiced on 12% of farms and on 7.6% of milk and beef cattle [48]. More than 600,000 cows and at least 30,000 mating bulls are kept for suckler cow husbandry, free ranging during several seasons of the year (BVL, personal communication, 2022). EU and federal programs endorse natural mating again. Thus, farmers producing cattle in conventional systems will also increase the use of mating bulls again. Additional state-run programs for conserving genetic resources guarantee the availability of a large genetic pool by keeping small herds of old farm animal breeds again [47]. Finally, the Zentrale Dokumentation Tiergenetischer Ressourcen in Deutschland (TGRDEU) of the BLE listed 27 endemic (German) livestock breeds but summed up 55 races kept in Germany [55]. The number of herdbook-covered breeds is actually higher than 40 (https://www.rind-schwein.de/brs-de/statistik-zahlen.html, assessed 15 February 2023). Although, the numbers of heads are exactly known due to the strict EU regulations for animal keeping, the total number of bovine races kept in the FRG was, and is, not exact due to the diversity of different interests in cattle breeding. Another important driver of change in agriculture is the public perception of the need for conservation of nature and the role of agriculture in this process. Contractual nature protection projects to keep smaller races on lean locations like nutrient-poor grassland, moors or reed lands are common practice and Heck cattle or buffaloes are kept semi-wild [56]. Formerly extinct bovine species like the European bison may become resident in Germany again and may be in contact with cattle herds kept on grassland during the summer time (https://www.wwf.de/themen-projekte/bedrohte-tier-und-pflanzenarten/wisente/wo-in-deutschland-wisente-leben-koennten, accessed on 26 April 2022). Wild, threatened bovines, but also threatened farm animal races, may be kept in zoos to ensure their survival in the future as the destruction of habitats moves on [57] (https://fzs.org/de/aktuelles/wisente-rueckkehr-der-wildrinder-16/, accessed on 15 February 2023). Currently, an area of research focus is the reduction of CH_4_ and N_2_O for the production of milk, as these are contributing to the greenhouse effect substantially and breeding will result in new races (https://www.bmel.de/SharedDocs/Praxisbericht/DE/forschungsprojekte/rinder-methan.html, accessed on 15 February 2023). An ongoing impact on the production of cattle is the demographic change. Jobs in animal production are not considered desirable and a massive labor shortage has resulted from this change in attitudes. In addition to the inefficiency of smaller farms, this led to a sharp decrease in the number of cattle farms by 25% to 108,000 within the last ten years. A farm is keeping 86.5 heads statistically. Hence, reality shows that usually more than 100 heads are kept per farm (66% of the stock), affecting herd health. An unpredictable driver of future structure change is the constantly accelerating climate change with steadily increasing mean annual temperatures and droughts in Middle Europe. The reduction of the acreage for fodder crops and yield will result in structural adaption of cattle production, whatever this will look like.

An unknown number of mating bulls is used for suckling calve production (more than 50,000 farms), organic farming (more than 5500 farms) and conventional farming due to the positive effects concerning intercalving time and pregnancy rate. Sexually mature bulls are usually used for less than two years in the herds because of their aggressiveness and for heredity reasons. Thus, these bulls can be found in the age groups ‘bulls one to two years’ and ‘bulls older than two years’ in official statistics. In this group alone, more than 90,000 bulls are registered.

It can be supposed, that at least one bull is used on every farm summing up to more than 50,000 bulls in use or to be used for mating. For special needs on the farms so-called ‘bulls for hire’ are also available. Although strict hygiene regulations are followed for mating bulls, including quarantine, these regulations will fail in BCG and trichomoniasis as these diseases do not cause clinical signs in bulls. An outbreak may run unnoticed until the first abortions occur and a high prevalence in cows and heifers must then be accepted. Efforts to monitor these diseases in exposed populations must therefore consider the experiences of the veterinary pioneers of AI. Applicable methods of sampling at slaughter and field abortion monitoring must be developed for early detection and well-timed countermeasures.

## 5. Conclusions

Recent ambitious efforts of German agricultural policies made for animal welfare, conservation of environment and genetic resources, and necessary structural adjustment must go hand in hand with control measures of veterinary public health. But is there a risk for a resurrection of bovine venereal diseases like BCG (and trichomoniasis) in Germany at all? Indeed, sporadic isolations of *Cfv* and outbreaks of BCG have been noted after 1990 in re-unified Germany, i.e., 39 notifications from 2006 to 2022 (2006, 6; 2007, 7; 2008, 9; 2009, 6; 2011, 1; 2012, 3; 2014, 3 and 2015, 2) (https://dserver.bundestag.de/btd/19/185/1918591.pdf, accessed on 15 February 2023) (Table 3).

This low prevalence faces a naive cow population and an increasing number of mating bulls no longer accessible for seamless monitoring as practiced at AI stations. In the absence of vaccines and effective testing capabilities, a short- and medium-term adjustment of the current strategy is needed.

## Figures and Tables

**Table 1:** Distribution of cattle breeds by type of production and cattle breeds with different ages in 2023 in Germany according to the Federal Agency for Agriculture and Food, The Federal Ministry of Food and Agriculture [48].

Cattle Breed	Total	Calves ≤8 m.	Calves >8 m.–1 y	Cattle >1–≤2 y	Cattle >2 y	Cows
**Dairy breeds (numbers × 1000)**						
Holstein-Schwarzbunt	4160.2	750.6	290.6	811.3	209.8	2097.9
Holstein-Rotbunt	466.1	82.8	33.8	94.0	30.1	225.4
Crossbreed	194.7	38.1	16.0	43.7	10.7	86.2
Angler	23.6	4.1	1.4	4.5	1.6	12.0
German Schwarzbunte (old breed)	18.9	3.6	1.7	4.3	1.9	7.3
Miscellaneous	31.1	5.3	2.4	6.5	2.6	14.3
**Beef breeds (numbers × 1000)**						
Crossbreed	564.8	126.9	64.4	152.5	42.1	179.0
Limousin	199.6	43.2	19.4	52.8	20.7	63.5
Charolais	108.4	22.7	8.1	28.0	10.7	38.9
Fleckvieh (Beef)	124.1	25.1	12.0	29.8	10.0	47.2
German Angus	116.4	26.9	8.6	28.4	11.3	41.2
Galloway	51.7	7.4	4.6	10.4	9.9	19.4
Highland	49.7	6.1	4.3	8.6	10.6	20.1
Buffaloes/Bisons	11.4	1.4	1.1	2.1	2.1	4.7
Miscellaneous	168.3	33.1	16.4	39.3	23.5	56.0
**Dual-purpose breeds (milk/meat) (numbers × 1000)**						
Fleckvieh	3094.7	688.0	331.3	824.5	183.0	1068.0
Braunvieh	298.6	48.3	21.5	62.2	21.8	144.8
Crossbreed	872.1	266.0	123.3	292.0	48.2	142.7
Dual-purpose Rotbunt	69.9	12.3	6.0	16.8	7.4	27.4
Other crossbreed	166.2	38.7	18.9	46.1	9.7	52.8
Vorderwälder	20.1	3.5	1.4	4.0	1.7	9.5
Gelbvieh	8.8	1.6	0.8	2.1	0.9	3.3
Miscellaneous	117.5	26.0	10.4	31.0	13.0	37.1

**Table 2:** Distribution of cattle in 16 German federal states in 2022–2023 according to the Federal Agency for Agriculture and Food, The Federal Ministry of Food and Agriculture [48].

Federal State	2022	2023	Difference
	*n* (%)	*n* (%)	
Baden-Wuerttemberg	912, 467 (8.3%)	903, 858 (8.26%)	−0.9
Bavaria	2, 867, 085 (26.1%)	2, 833, 433 (25.90%)	−1.2
Berlin	784 (0.01%)	741 (0.007%)	−5.5
Brandenburg	448, 309 (4.10%)	446, 303 (4.08%)	−0.4
Bremen	8, 274 (0.075%)	8, 269 (0.076%)	−0.1
Hamburg	5, 861 (0.053%)	5, 793 (0.053%)	−1.2
Hesse	391, 587 (3.56%)	392, 436 (3.59%)	+0.2
Mecklenburg-Western Pomerania	458, 837 (4.17%)	461, 394 (4.22%)	+0.6
Lower-Saxony	2, 350, 584 (21.37%)	2, 352, 926 (21.51%)	+0.1
North Rhine-Westphalia	1, 272, 505 (11.57%)	1, 260, 319 (11.52%)	−1.0
Rhineland-Palatinate	299, 575 (2.72%)	298, 020 (2.72%)	−0.5
Saarland	39, 575 (0.36%)	39, 355 (0.36%)	−0.6
Saxony	435, 024 (3.96%)	435, 284 (3.98%)	+0.1
Saxony-Anhalt	278, 086 (2.53%)	276, 488 (2.53%)	−0.6
Schleswig-Holstein	950, 534 (8.64%)	949, 171 (8.68%)	−0.1
Thuringia	277, 876 (2.53%)	273, 008 (2.50%)	−1.8
**Total**	10, 996, 963	10, 936, 798	−0.5

**Table 3:** Number of outbreaks of BGC in German federal states in the years 2006–2022 according to the Animal Disease News System (TSN).

	2006	2007	2008	2009	2010	2011	2012	2013	2014	2015	2016	2017	2018	2019	2020	2021	2022
Baden-Wuerttemberg	2	1	-	-	-	-	-	-	-	-	-	-	-	-	-	-	-
Bavaria	4	6	8	3			3	2	2	1	-	-	-	-	-	-	-
Brandenburg	-	-	-	-	-	-		1	-	-	-	-	-	-	-	-	-
Lower-Saxony	-	-	-	3	-	-	-	-	-	-	-	-	-	-	-	-	-
North Rhine-Westphalia	-	-	-		-	-	-	-	-	1	-	-	-	-	-	-	-
Rhineland-Palatinate	-	-	-	-	-	1	-	-	-	-	-	-	-	-	-	-	-
Saxony	-	-	1	-	-		-	-	-	-	-	-	-	-	-	-	-
**Total**	**6**	**7**	**9**	**6**	**-**	**1**	**3**	**3**	**2**	**2**	**-**	**-**	**-**	**-**	**-**	**-**	**-**

## Data Availability

The datasets used and/or analyzed during the current study are presented in the manuscript and available from the corresponding authors on reasonable request. The corresponding authors (H.E.-A. and H.N.) can be contacted for information regarding the dataset.
